# Conservative oxygen therapy for critically ill patients: a meta-analysis of randomized controlled trials

**DOI:** 10.1186/s40560-021-00563-7

**Published:** 2021-07-22

**Authors:** Xiao-Li Chen, Bei-Lei Zhang, Chang Meng, Hui-Bin Huang, Bin Du

**Affiliations:** 1 Department of Critical Care Medicine, the First Affiliated Hospital of Fujian Medical University, Fuzhou, China; 2 Department of Critical Care Medicine, Beijing Tsinghua Changgung Hospital, School of Clinical Medicine, Tsinghua University, Beijing, 102218 China; 3 Medical ICU, Peking Union Medical College Hospital, Peking Union Medical College and Chinese Academy of Medical Sciences, 1 Shuai Fu Yuan, Beijing, 100730 China

**Keywords:** Conservative oxygen strategy, Mortality, Critically ill, Meta-analysis

## Abstract

**Objective:**

Conservative oxygen strategy is recommended in acute illness while its benefit in ICU patients remains controversial. Therefore, we sought to conduct a systematic review and meta-analysis to examine such oxygen strategies’ effect and safety in ICU patients.

**Methods:**

We searched PubMed, Embase, and the Cochrane database from inception to Feb 15, 2021. Randomized controlled trials (RCTs) that compared a conservative oxygen strategy to a conventional strategy in critically ill patients were included. Results were expressed as mean difference (MD) and risk ratio (RR) with a 95% confidence interval (CI). The primary outcome was the longest follow-up mortality. Heterogeneity, sensitivity analysis, and publication bias were also investigated to test the robustness of the primary outcome.

**Results:**

We included seven trials with a total of 5265 patients. In general, the conventional group had significantly higher SpO_2_ or PaO_2_ than that in the conservative group. No statistically significant differences were found in the longest follow-up mortality (RR, 1.03; 95% CI, 0.97–1.10; *I*^2^=18%; *P*=0.34) between the two oxygen strategies when pooling studies enrolling subjects with various degrees of hypoxemia. Further sensitivity analysis showed that ICU patients with mild-to-moderate hypoxemia (PaO_2_/FiO_2_ >100 mmHg) had significantly lower mortality (RR, 1.24; 95% CI, 1.05–1.46; *I*^2^=0%; *P*=0.01) when receiving conservative oxygen therapy. These findings were also confirmed in other study periods. Additional, secondary outcomes of the duration of mechanical ventilation, the length of stay in the ICU and hospital, change in sequential organ failure assessment score, and adverse events were comparable between the two strategies.

**Conclusions:**

Our findings indicate that conservative oxygen therapy strategy did not improve the prognosis of the overall ICU patients. The subgroup of ICU patients with mild to moderate hypoxemia might obtain prognosis benefit from such a strategy without affecting other critical clinical results.

**Supplementary Information:**

The online version contains supplementary material available at 10.1186/s40560-021-00563-7.

## Introduction

Supplemental oxygen is an essential therapy for patients. In the intensive care unit (ICU) setting, critically ill patients with or at risk for impaired pulmonary gas exchange always receive oxygen therapy to avoid hypoxemia [1]. Clinicians may tend to feel reassured when patients’ oxygen saturation approaches 100%. However, increasing data have shown that exposure to high levels of inspired oxygen may relate to many adverse events, such as acute lung injury, interstitial fibrosis, and bronchitis [2, 3]. Besides, hyperoxia may also cause cardiovascular, cerebrovascular, and systemic peripheral vasoconstriction, and decreased cardiac output, leading to ischemia and hypoxia in various organs [4–6]. Thus, a conservative oxygen strategy has been proposed [7].

In 2016, Girardis et al. conducted a randomized controlled trial (RCT) to compare conservative oxygen strategy with conventional oxygen therapy in ICU patients. They found that a conservative strategy (maintain PaO_2_ between 70 and 100 mmHg or arterial oxyhemoglobin saturation [SpO_2_] between 94 and 98%) had lower mortality and more ventilator-free days [8]. In the same year, a published meta-analysis that included 25 RCTs with 16,037 acutely ill patients supported implementing such a conservative oxygen strategy [1]. However, the meta-analysis had significant heterogeneity in disease categories, including stroke, trauma, sepsis, cardiac, and emergency surgery. Moreover, stroke and myocardial infarction contributed more than 90% of the included patients in this meta-analysis, while only two ICU studies, accounting for 4% of the overall patients, were included.

Additionally, two meta-analyses about the same topics only focusing on ICU patients were published in 2019 [9, 10] and showed that conservative oxygen therapy significantly reduced short-term mortality and the incidence of organ dysfunction. However, the inclusion of few, relatively small studies with different study designs might result in significant heterogeneity. Therefore, considering most ICU patients have more severe hypoxemia, using conservative oxygen therapy in these patients remains controversial.

Several studies comparing different oxygen strategies in ICU patients have recently been published, and some of these studies had a relatively large sample size [11–13]. Therefore, to address the limitations of previous analyses, we sought to conduct a systematic review and meta-analysis by pooling available RCTs to examine these two oxygen strategies’ effect and safety in this patient population.

## Methods

We conducted the current systematic review and meta-analysis following the PRISMA guidance [14] (Additional file 1). The protocol has been registered on the International Platform of Registered Systematic Review and Meta-analysis Protocols database (INPLASY202070044) and is available in full on inplasy.com (https://doi.org/ 10.37766/inplasy2020.7.0044).

### Search strategy

Two authors (X-L C and CM) independently searched potentially relevant studies in PubMed, Embase, and the Cochrane database from inception to Feb 15, 2021. The details in the search strategy are summarized in Additional file 2. Our study was limited to RCTs, and no language restriction was applied. Reference lists of included articles and other meta-analyses were also reviewed.

### Selection criteria

We included RCTs that focused on adult ICU patients receiving either conservative oxygen strategy or conventional oxygen strategy. Each study’s authors determined the specific definitions of both oxygen therapy strategies. Predefined outcomes included mortality rate, length of stay in ICU and hospital, duration of mechanical ventilation (MV), and adverse events during treatment. We excluded studies enrolling patients <18 years old, suffering the risk of ischemia or hypoxic encephalopathy (i.e., stroke, myocardial infarction, and cardiac arrest), and receiving palliative care, and publications only in abstract form or meeting reports. We contacted the authors if associated data from their studies were required.

### Data extraction and quality assessment

Two authors (X-LC and CM) independently extracted data from the included studies, such as the first author, year of publication, setting, country, sample size, study design, setting, treatment protocols, comparator, and severity of illness as well as all predefined outcomes. The included studies’ quality was evaluated using the risk of bias tool recommended by the Cochrane Collaboration [15]. We assigned a value of high, unclear, and low to the following items: sequence generation, allocation concealment, blinding, incomplete outcome data, selective outcome reporting, and other sources of bias. We identified and resolved the discrepancies through discussion.

### Statistical analysis

The primary outcome was the longest follow-up mortality. To explore the robustness and the influence of hypoxemia severity on our primary outcome, we performed sensitivity analyses based on PaO_2_/FiO_2_ levels (i.e., mild, moderate, and severe hypoxemia, defined as PaO_2_/FiO_2_ of >200 mmHg, 100–200 mmHg, and <100 mmHg, respectively). We also evaluated other mortality at different study periods (i.e., stay in ICU, or 30 days, 60 days, 90 days after recruitment), if available. Secondary outcomes included oxygen exposures (i.e., SpO_2_, PaO_2_, FiO_2_, and PaO_2_: FiO_2_), duration of MV, length of stay in ICU and hospital, Sequential Organ Failure Assessment (SOFA) scores after treatment, and adverse events.

The results from all relevant studies were merged to estimate the pooled risk ratio (RR) and associated 95% confidence intervals (CIs) for dichotomous outcomes. For the continuous outcomes, mean differences (MD) and 95% CIs were estimated as effective. Some included RCTs reported median as the measure of treatment effect, with interquartile range (IQR). We estimated the mean from median and standard deviations (SD) from IQR using the methods described in the previous studies [16]. Heterogeneity was tested with *I*^2^ statistics. An *I*^2^ < 50% indicates insignificant heterogeneity, and we used a fixed-effect model accordingly, whereas we chose a random-effect model in cases of significant heterogeneity (*I*^2^ > 50%) [17]. Publication bias was evaluated by visually inspecting funnel plots when at least ten studies were included in this meta-analysis. A *P*-value of < 0.05 was considered statistically significant. All statistical analyses were performed by Review Manager Version 5.3.

## Result

### Study selection

**Fig. 1:**
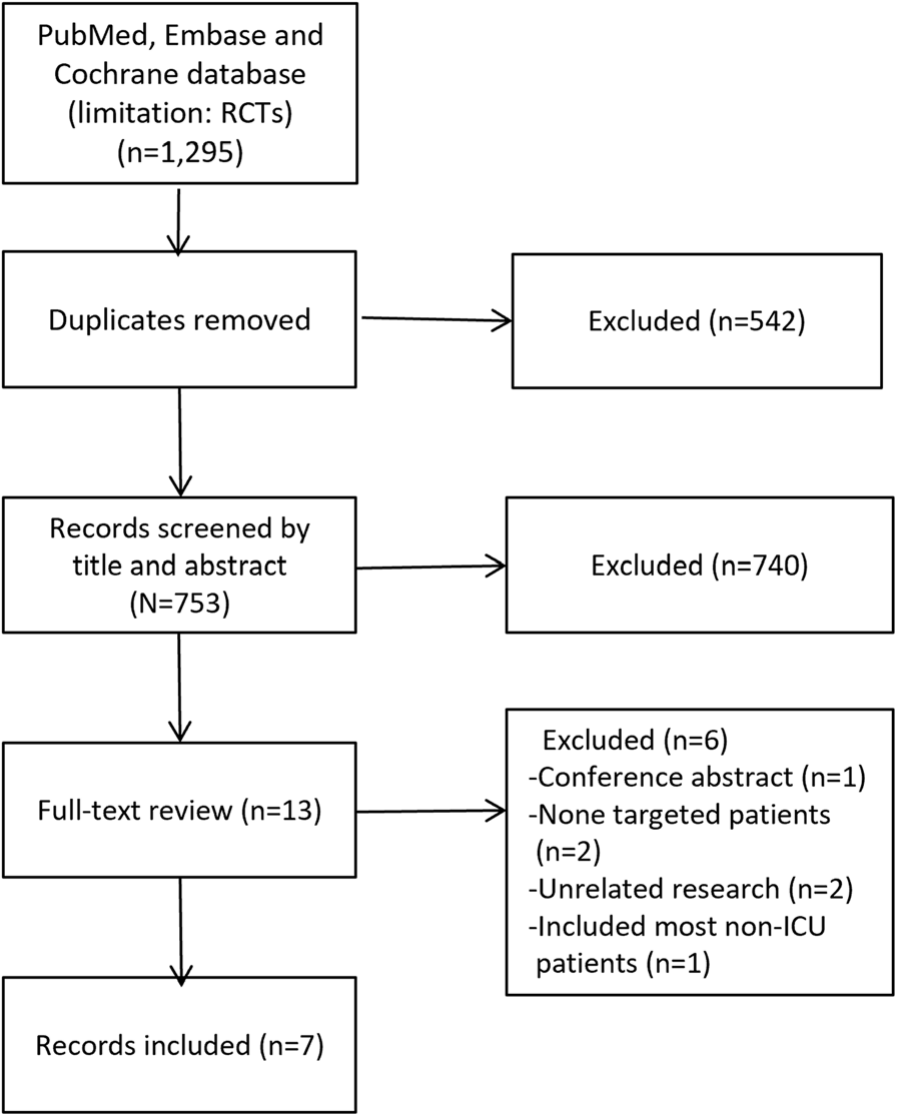
Selection process for RCTs included in the meta-analysis

The electronic search identified 1295 relevant studies. Of these, 13 full-text studies were read for potential eligibility. Based on the full-text evaluation, we excluded five studies summarized in Additional file 3 with exclusion reasons. Finally, we included 7 RCTs, with a total of 5265 patients, in this systemic review and meta-analysis [2, 8, 11–13, 18, 19] (Fig. 1).

### Study characteristics and quality

The characteristics of the included RCTs are described in Table 1. The definitions of inclusion and exclusion criteria and oxygen therapy regimen for patients are summarized in Additional file 4. These studies were published between 2015 and 2021, with sample sizes ranging from 104 to 2928 cases. Five of the 7 RCTs were multicenter studies [2, 11, 12, 18, 19]. The hypoxemia severity of recruiting patients varied among the included trials, with 4 RCTs included patients without hypoxemia severity limited [11, 12, 18, 19], while 2 RCTs excluded patients with PaO_2_/FiO_2_ <100 mmHg [2, 13], and 1 RCT excluded patients with PaO_2_/FiO_2_ <150 mmHg [8]. Three RCTs were terminated early for safety reasons, low likelihood of a significant difference between the groups, difficulty in patient recruitment, or significant difference between the two groups [2, 8, 11].

**Table 1:** Characteristics of the studies included in current systemic review and meta-analysis

Study	Study design	*N*	Included patients	Patient characteristics (conventional/conservative)				Conservative oxygen regimen	Conventional oxygen regimen	Follow-up (days)
				Severe ARF included	Age, mean, (year)	Disease severity	Patients with MV (%)			
Schjørring et al. [19]	PR, UB, MC	1441/1447	Aged ≥18 admitted to ICU with hypoxemic respiratory failure	Yes	70/70	SOFA 9/9	71/72	PaO_2_ target 60 mmHg	PaO_2_ target 90 mmHg	90
Barrot et al. [11]	PR, UB, MC	102/103	Adult patients receiving MV ≤12 h for ARDS	Yes	64/64	SOFA 9/9 SAPS III 68/67	100/100	PaO_2_ target 55–70 mmHg; SpO_2_ target 88–92%	PaO_2_ target 90–105 mmHg; SpO_2_ ≥96%	90
Yang et al. [13]	PR, SB, SC	114/100	Aged ≥18 admitted to ICU with expected ICU LOS ≥ 72 h	PiO_2_ <100 were excluded	60/56	APACHE II 17/17	84/83	SpO_2_ target 90–95%; FiO_2_ as low as possible	SpO_2_ target 96–100%; FiO_2_ no lower than 30%	28
Mackle et al. [12]	PR, SB, MC	501/499	Age ≥18 admitted to ICU and expected to receive MV ≥48 h	Yes	68/65	APACHE II 22/19	100/100	SpO_2_ target 90–97%	No specific limiting FiO_2_ or SpO_2_	250
Asfar et al. [2]	PR, UB, MC	219/223	Aged ≥18 with septic shock who were on MV	PiO_2_ <100 were excluded	58/62	SOFA 10/10 SAPS III 72/73	100/100	SpO_2_ target 88–95%	FiO_2_ of 1.0 for 24 h	90
Girardis et al. [8]	PR, UB, SC	244/236	Age ≥18 admitted to ICU with expected ICU LOS ≥72 h	PiO_2_ <150 were excluded	65/63	SAPS II 39/37	68/66	SpO_2_ target 94–98% or PaO_2_ target 70–100 mmHg	FiO_2_ ≥0.4, PaO_2_ up to 150 mmHg, SpO_2_ of 97–100%	60
Panwar et al. [18]	PR, UB, MC	51/53	Age ≥18 admitted to ICU with expected MV time ≥24 h	Yes	62/62	APACHE III 70/80 SOFA 7/8	100/100	SpO_2_ target 88–92%	SaO_2_ target ≥96%	90

*APACHE*, Acute Physiology, Age, and Chronic Health Evaluation; *ARDS*, acute respiratory distress syndrome; *FiO*_*2*_, fraction of inspiratory oxygen; *ICU*, intensive care unit; ^*a*^*ITT*, intention-to-treat analysis; *LOS*, length of stay; *MV*, mechanical ventilation; *MC*, multi-centers; *PaO*_*2*_, partial pressure of arterial oxygen; *PiO*_*2*_, PaO_2_/FiO_2_; *PR*, prospective; *SAPA*, Simplified Acute Physiology score; *SC*, single center; *SB*, single-blind; *SOFA*, Sequential Organ Failure Assessment; *SpO*_*2*_, pulse oxygen saturation; *SARF*, severe acute respiratory failure; *UB*, unblinded

The Cochrane risk of bias score for each citation varied across the studies (Additional file 5). All included RCTs were at high risks because of unblinding design. We did not assess the publication bias because of the limited number (< 10) of each analysis’s studies.

### Primary outcome

**Fig. 2:**
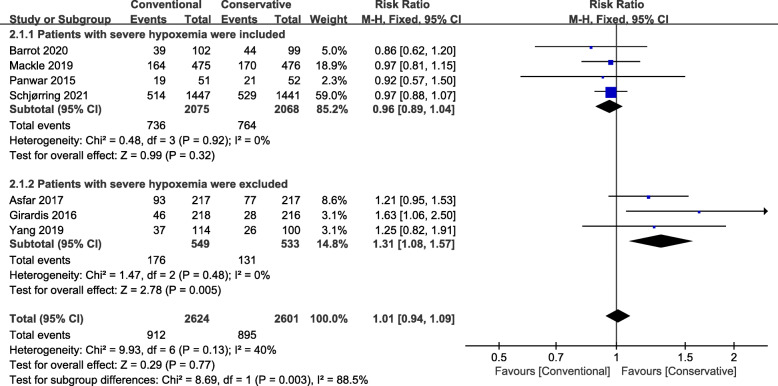
Forest plot showing the effect of conservative versus conventional oxygen therapy on the mortality rate in ICU patients with severe hypoxemia were included (2.1.1) and in ICU patients with severe hypoxemia were excluded (2.1.2)

All 7 RCTs reported the longest follow-up mortality, and the pooled analysis showed similar mortality rate between conservative and conventional groups (7 RCTs, *N*=5225; RR, 1.01; 95% CI, 0.94–1.09; *I*^2^=40%; *P*=0.77) [2, 8, 11–13, 18, 19] (Fig. 2). Further excluding any single study did not significantly change the overall combined RR, ranging from 1.02 (95% CI, 0.91–1.15; *P* = 0.71) to 1.12 (95% CI, 0.95–1.32; *P* = 0.19).

Subsequently, we performed a predefined sensitivity analysis. Among the included RCTs, four included ICU patients with various levels of hypoxemia [11, 12, 18, 19], and three studies [2, 8, 13] excluded patients with severe hypoxemia from their inclusion criteria. Therefore, we pooled these two types of studies with or without severe hypoxemia (PaO_2_/FiO_2_ <100 mmHg) separately. When the analysis was limited to those RCTs that included all levels of hypoxemia of patients, the combined RR remained unchanged (4 RCTs, *N*=4143; RR, 0.96; 95% CI, 0.89–1.04; *I*^2^=0%; *P*=0.32) (Fig. 2 (2.1.1)). When only combining RCTs that excluded patients with severe hypoxemia, we found that conservative oxygen strategies can significantly decrease short-term mortality (3 RCTs, *N*=1082; RR, 1.31; 95% CI, 1.08–1.57; *I*^2^=0%; *P*=0.005) (Fig. 2 (2.1.2)). These findings were also confirmed in other study periods (Table 2).

**Table 2:** Summary of sensitivity analyses of mortality rates of primary outcome

Research periods	Mortality rates of conservative oxygen strategy compared with conventional oxygen strategy		
	All included studies	Studies of patients with severe RF	Studies of patients without severe RF
Longest follow-up	7 RCTs, *N*=5225; 1.01 (0.94–1.09) [2, 8, 11–13, 18, 19]	4 RCTs, *N*=4143; 0.99 (0.92–1.07) [11, 12, 18, 19]	3 RCTs, *N*=1082; 1.24 (1.05–1.46) [2, 8, 13]
Stay in ICU	5 RCTs, *N*=1386; 1.19 (0.89, 1.60) [2, 8, 11, 13, 18]	2 RCTs, *N*=304; 0.78 (0.55–1.11) [11, 18]	3 RCTs, *N*=1082; 1.41 (1.17–1.70) [2, 8, 13]
30 days after recruitment	5 RCTs, *N*=4171; 1.10 (0.90–1.34) [2, 8, 11, 13, 19]	2 RCTs, *N*=3089; 0.95 (0.85–1.07) [11, 19]	3 RCTs, *N*=1082; 1.29 (1.07–1.55) [2, 8, 13]
60 days after recruitment	4 RCTs, *N*=3957; 1.06 (0.90, 1.24) [2, 8, 11, 19]	2 RCTs, *N*=3089; 0.97 (0.89–1.06) [11, 19]	2 RCTs, *N*=868; 1.22 (1.02–1.46) [2, 8]
90 days after recruitment	5 RCTs, *N*=4585; 1.00 (0.93–1.07) [2, 11, 12, 18, 19]	4 RCTs, *N*=4151; 0.98 (0.91–1.06) [11, 12, 18, 19]	-

*RF*, respiratory failure; *ICU*, intensive care unit

### Secondary outcomes

All the included RCTs reported the oxygen exposures after treatment and suggested that the conventional group had significantly higher SpO_2_ and PaO_2_ than that in the conservative group (Table 3). Four RCTs [2, 8, 12, 18] reported the outcome of MV-free days, and pooled data showed no significant difference between the two groups (4 RCTs, *n*=707, SMD, −0.15 days; 95% CI, −0.48 to 0.17, *I*^2^=91%; *P*=0.36). The length of stay in the ICU (4 RCTs [2, 8, 12, 18], *n*=1936, MD, 0.17 days; 95% CI, −0.36 to 0.69, *I*^2^=33%; *P*=0.53) and hospital [8, 12, 18] (3 RCTs, *n*=1466, MD, −0.53 days; 95% CI, −2.03 to 0.98, *I*^2^=0%; *P*=0.49) was also similar. ∆SOFA score was evaluated by 3 RCTs [2, 11, 18] and showed no difference between groups (3 RCTs, *n*=1466, MD, −0.53 days; 95% CI, −2.03 to 0.98, *I*^2^=0%; *P*=0.49). As to adverse events, pneumonia (3 RCTs [2, 8, 11], *n*=1069; RR, 0.92; 95% CI, 0.72 to 1.18, *I*^2^=33%; *P*=0.52), mesenteric ischemia (3 RCTs [2, 11, 19], *n*=3545, RR, 1.15; 95% CI, 0.73 to 1.19, *I*^2^=47%; *P*=0.55), and stroke (3 RCTs [11, 12, 19], *n*=4076, RR, 0.93; 95% CI, 0.53 to 1.63, *I*^2^=15%; *P*=0.79) were the most frequently reported among the included studies and were comparable between the groups. The details in other adverse events are summarized in Additional file 6.

**Table 3:** The predefined oxygen exposures during study period between the two oxygen regimens among the included studies

Study/year	Oxygen exposures (conservative group vs. conventional group)			
	SpO_**2**_ (%)	PaO_**2**_ (mmHg)	PaO_**2**_:FiO_**2**_ (mmHg)	FiO_**2**_ (%)
Schjørring et al., 2021 [19]	93 (92–94) vs. 96 (95–97), *P* <0.05	71 (67–77) vs. 93 (87–99), *P* <0.05		43 (34–54) vs. 56 (46–71), *P* <0.05
Barrot et al., 2020 [11]	−3.8 (95% CI, −4.3 to −3.2)	−28.1 (95% CI, −38.4 to −17.7)	NR	−15.5 (95% CI, −19.1 to −12.0)
Yang et al., 2018 [13]	95.7±2.3 vs. 98.2±1.8, *P*<0.001	84 (71–99) vs. 98 (79–116), *P*=0.060	NR	33 (25–42) vs. 42 (36–50)
Girardis et al., 2016 [8]	NR	87 (79–97) vs. 102 (88–116), *P*<0.001	50±97 vs. 21±102, *P*=0.15	0.36 (0.30–0.40) vs. 0.39 (0.35–0.42), *P*<0.001
Panwar et al., 2015 [18]	93.4 (92.9–93.9) vs.97 (96.5–97.5), *P* <0.001	70 (68–73) vs. 92 (89–96), *P* <0.001	NR	0.26 (0.25-0.28) vs. 0.36 (0.34-0.39), *P*<0.001
Asfar et al., 2017 [2]	NR	*P*<0.0001	*P*=0.0039	NR
Mackle et al., 2019 [12]	The mean FiO_2_ and PaO_2_ during the first 10 days of MV, and the lowest and highest FiO_2_ and PaO_2_ values until day 28 were lower in the conservative group			

Data expressed as median (IQR) or mean±standard deviation

Abbreviations: *FiO*_*2*_, fraction of inspiratory oxygen; *ICU*, intensive care unit; *MV*, mechanical ventilation; *NR*, not reported; *PaO*_*2*_, partial pressure of arterial oxygen; *SpO*_*2*_, pulse oxygen saturation

## Discussion

In this update systemic review and meta-analysis, we investigated the safety and effectiveness of conservative oxygen strategy in ICU patients. Our findings indicated that conservative oxygen therapy strategy did not improve the prognosis of the overall ICU patients. However, further sensitivity analysis showed that patients with mild-to-moderate hypoxemia and conservative strategy had a significantly lower mortality rate, without affecting other important clinical outcomes such as MV-free days, length of stay in ICU and hospital, ∆SOFA, and adverse events.

### Our results in relation to previous reviews

Two recently published meta-analyses reported that conservative oxygen therapy significantly improved survival in ICU patients [9, 10]. However, interpretation of the results from Hirase et al. is limited because their meta-analysis [9] was based on only four trials, including 742 cases, and two of these trials were observational studies [20, 21], thus leading to potential selection bias. The same applies to the meta-analysis by Liu et al. [10]. The study also pooled four trials; one was an observational study [20], and one RCT was in abstract form only [22]. To address these shortcomings, we included only RCTs focusing on ICU patients in our meta-analysis. In addition to the previously included RCTs [2, 8], we added five more recently published RCTs [11–13, 18, 19] with a more statistical power of 5265 patients to examine our primary outcome. Further sensitivity analysis suggested that patients with different hypoxemia severity might contribute to the heterogeneity in the present meta-analysis, whereas sensitivity analysis basing hypoxemia severity resolved the issue of heterogeneity among the included studies.

Another most recently published meta-analysis by Zhao et al. [23] investigated the association between different oxygenation goals and the prognosis in critically ill mechanical ventilation patients. The authors found a worse prognosis for patients with liberal (PaO_2_>150 mmHg) or far more conservative targets (PaO_2_=55–70 mmHg) had a worse prognosis than the moderate (PaO_2_=90–150 mmHg). Though direct comparison was unavailable with our study because of the differences in study design and oxygenation classification, the meta-analysis suggested that different oxygenation goals might potentially lead to different mortalities in ventilated ICU patients.

### Explain the results of our research

We found that ICU patients with mild-to-moderate hypoxemia can benefit from conservative oxygen therapy strategies. This finding is consistent with that of acutely ill patients’ findings in previous study (for example, patients with myocardial infarction and stroke) [8]. Conservative oxygen therapy can avoid the harm caused by hyperoxemia. Theoretically, during the ischemia-reperfusion process of ICU patients, hyperoxemia will affect the synthesis of ATP and promote the production of oxygen free radicals [24]. Simultaneously, hyperoxemia can cause superoxide and oxygen-free radical-mediated lung damage, leading to pulmonary interstitial fibrosis, atelectasis, bronchitis, alveolar protein leakage, and neutrophil infiltration [25].

Previous studies suggested that exposure to pure oxygen for about 6 to 25 h might cause clinical and histological changes such as bronchitis and alveolitis [26]. In the study by Suzuki et al. [27], the authors reported that the conservative strategy decreased the median total amount of oxygen delivered during MV by about two-thirds; it could reduce radiation-related atelectasis, weaning from the mandatory ventilation mode, and switch to the spontaneous breathing mode earlier. A French retrospective observational study [28] suggested that hyperoxemia, defined as PaO_2_ >120 mmHg, is independently associated with VAP (OR=1.89, 95% CI 1.23, 2.89). Moreover, the longer the patients were exposed to hyperoxia, the higher the incidence of VAP. Also, in clinical practice, a lower oxygenation target can lower the demand for respiratory support intensity of mechanically ventilated patients and reduce the occurrence of ventilator-related lung injury to a certain extent.

On the other hand, our results did not support the application of such a strategy for ICU patients with severe hypoxemia. Theoretically, these patients had more severe gas-exchange impairments and refractory hypoxemia, requiring higher intensity respiratory support [29]. As shown in the present meta-analysis, 83% (4316/5225) of the included patients are receiving MV [2, 8, 11–13, 18, 19]. The average PaO_2_/FiO_2_ ratio of patients in some included studies is only about 100 mmHg [11, 19]. In such scenarios, clinicians should pay more attention to oxygen delivery and oxygen utilization. Additionally, some advanced respiratory support techniques may be required for such a patient population, such as optimal PEEP titration, prone position ventilation, sedative anesthetic, muscle relaxant application, and even extracorporeal membrane oxygenation [7]. Data from ARDS patients have demonstrated that lower oxygenation levels are associated with poorer long-term neuropsychological and cognitive outcomes [30]. Thus, from a pathophysiological perspective, a conservative oxygen strategy does not seem appropriate at this time. Potential impairment due to high oxygen levels may not be a significant consideration for treatment influences in severe acute respiratory failure patients.

### Current literature and future research

It is worth noting that there is currently no uniform standard for the oxygen titration setting of conservative oxygen therapy strategies. First, all the RCTs used SpO_2_ for monitoring. However, in some trials, the actual measured SpO_2_ differences between the conservative and the conventional groups were small (median of 2–4%) [11, 13, 18], and it seems unlikely that significant differences in mortality between the groups could be obtained. Second, some critical situations in ICU patients, such as severe hypoperfusion, can affect SpO_2_ measurement accuracy [31]. SpO_2_ is also much more dependent upon acid base and many other items. Additionally, though also used as oxygen titration in some trials, PaO_2_ cannot be continuously monitored and provide adequate monitoring and timely adjustments. Future trials will have to address how to set and implement a specific standard to reduce the defects of oxygenation target monitoring.

In addition, in patients with ARDS, its high degree of heterogeneity may be a potential cause of treatment failure [32, 33]. Researchers have currently proposed two different clinical subtypes of ARDS based on large-scale RCTs and biomarker changes [31]. Compared to subtype 1, subtype 2 is characterized by stronger inflammatory responses; longer hospital stays, MV time, and organ failure duration; and a poor prognosis. Such patients require higher PEEP support and restrictive fluid management [34]. Studies also found that ARDS patients with diffuse alveolar damage (DAD) were more likely to suffer severe oxygenation and worsened respiratory compliance, and often die of refractory hypoxemia [35]. However, although the included RCTs enrolled ARDS patients, none of them focused on screening ARDS subtypes. It remains unclear whether patients with different subtypes of ARDS respond differently to conservative oxygen therapy strategies.

Notably, some of the included studies reported finding more ischemic disorders such as intestinal ischemia [11] or myocardial ischemia [19] in the conservative oxygen group. These findings suggest the necessity of using microcirculatory assessment tools to guide monitoring during conservative treatment, in addition to macro-circulation. For instance, increased lactate can be early signs of mesenteric ischemia [36], jugular SvO_2_ as the cerebral oxygenation measurement to measure tissue oxygen saturation in the brain [37], and the sublingual microcirculation may well reflect the state of the tissue microcirculation [38]. These microcirculatory assessment tools might be helpful for early detection and prevention of hypoxia-related complications.

### Strengths and limitations

Our study has some limitations. First, most included studies were unblinded [2, 8, 11, 18], which would more likely result in performance bias. However, this is determined by the study design; clinicians require dynamically adjusting the oxygenation target during the study period. Second, 3 trials had unexpected early termination of their researches and might increase the likelihood of overestimating [2, 8, 11]. Third, there was overlap among included trials concerning the range of SpO_2_ targets between conservative and conventional oxygen groups, and potentially compromises our findings’ robustness. Fourth, the uneven distribution of severe hypoxemia among included studies might also exert a prognostic value. We planned to perform subgroup analyses to explore studies based on such diversities, which was hampered by insufficient data. Finally, most of the included studies did not evaluate the long-term neurological function and complications, requiring more attention in future research.

## Conclusion

In summary, our findings indicate that conservative oxygen therapy strategy did not improve the prognosis of the overall ICU patients. ICU patients with mild to moderate respiratory failure might obtain prognosis benefit from such a strategy without affecting other critical clinical results. Further studies are needed to identify our findings.

## Supplementary Information

**Additional file 1.** PRISMA checklist.

**Additional file 2.** Search.

**Additional file 3.** Table: Studies needed for full-reviewed but not included in the current meta-analysis.

**Additional file 4.** Fig: Definition of inclusion and exclusion criteria for patient population and regimens of conservative and conventional oxygen.

**Additional file 5.** Fig: Cochrane risk of bias.

**Additional file 6.** Table: Adverse events.

## Data Availability

All data generated or analyzed during this study are included in this published article.

## Abbreviations

CI: Confidence interval
FiO_2_: Fraction of inspiratory oxygen
ICU: Intensive care unit
IQR: Interquartile range
MD: Mean difference
MV: Mechanical ventilation
PaO_2_: Partial pressure of arterial oxygen
RR: Risk ratio
RCTs: Randomized controlled trials
SOFA: Sequential organ failure assessment
SpO_2_: Arterial oxyhemoglobin saturation
