# Supplementary material for: Conservative oxygen therapy for critically ill patients: a meta-analysis of randomized controlled trials
Source: J Intensive Care. 2021 Jul 22;9:47. doi: 10.1186/s40560-021-00563-7 (PMC8295978; doi:10.1186/s40560-021-00563-7)
Supplement: Supplementary file 2 — Additional file 2. Search. [file 40560_2021_563_MOESM2_ESM.docx]

**Additional file 2**

**Search Strategy：**

**Database: PubMed, Embase, Cochrane library；**

**Search completed on 15th Feb 2021.**

=====================================================================================================================

**PubMed**

(((((((conservative oxygen[Title/Abstract]) OR (Hyperoxia[Title/Abstract])) OR (Liberal oxygen[Title/Abstract])) OR (Conventional Oxygen[Title/Abstract])) OR (hypoxemic[Title/Abstract])) OR (lower-oxygenation[Title/Abstract])) AND ((randomized controlled trial [pt] OR controlled clinical trial [pt] OR randomized [tiab] OR placebo [tiab] OR clinical trials as topic [mesh: noexp] OR randomly [tiab] OR trial [ti]) NOT (animals [mh] NOT humans [mh]))) AND (("critical care"[mesh]) OR (((critical care[Title/Abstract]) OR (critically ill[Title/Abstract])) OR (intensive care[Title/Abstract])))

**-------------------------------------------------------------------------------------------------------------------------------------------------------------------------------------------------------**

**Embase**

#15. #1 AND #6 AND #14

#14. #7 OR #8 OR #9 OR #10 OR #11 OR #12 OR #13

#13. 'lower-oxygenation':ab,ti

#12. 'hypoxemic':ab,ti

#11. 'liberal oxygen':ab,ti

#10. 'hyperoxia':ab,ti

#9. 'conventional oxygen':ab,ti

#8. 'conservative oxygen':ab,ti

#7. 'conservative oxygen'

#6. #2 OR #3 OR #4 OR #5

#5. 'intensive care':ab,ti

#4. 'critical care':ab,ti

#3. 'critically ill':ab,ti

#2. 'critically ill'/exp

#1. 'clinical trial'/exp OR 'clinical trial' OR 'randomization'/exp OR 'randomization' OR 'single blind procedure'/exp OR 'single blind procedure' OR 'double blind procedure'/exp OR 'double blind procedure' OR 'randomized controlled trial'/exp OR 'randomized controlled trial' OR 'crossover procedure'/exp OR 'crossover procedure' OR 'placebo'/exp OR 'placebo' OR 'prospective studies'/exp OR 'prospective studies' OR ('randomi?ed controlled' NEXT/1 trial*) OR RCT OR 'randomly allocated' OR 'allocated randomly' OR 'random allocation'/exp OR 'random allocation' OR (allocated NEAR/2 random) OR (single NEXT/1 blind*) OR (double NEXT/1 blind*) OR ((treble OR triple) NEAR/1 blind*) OR placebo* #18 #14 OR #15 OR #16 OR

---------------------------------------------------------------------------------------------------------------------------------------------------------------------------------------------------------

**Cochrane library**

#1 ("intensive care"):ti,ab,kw (Word variations have been searched)

#2 ("critically ill"):ti,ab,kw (Word variations have been searched)

#3 ("critical care"):ti,ab,kw (Word variations have been searched)

#4 ("critical illness"):ti,ab,kw (Word variations have been searched)

#5 #1 OR #2 OR #3 OR #4

#6 ("lower-oxygenation"):ti,ab,kw (Word variations have been searched)

#7 ("hypoxemic"):ti,ab,kw (Word variations have been searched)

#8 ("liberal oxygen"):ti,ab,kw (Word variations have been searched)

#9 ("hyperoxia"):ti,ab,kw (Word variations have been searched)

#10 ("conventional oxygen"):ti,ab,kw (Word variations have been searched)

#11 ("conservative oxygen"):ti,ab,kw (Word variations have been searched)

#12 #6 OR #7 OR #8 OR #9 OR #10 OR #11

#13 #5 AND #12
