# Supplementary material for: Conservative oxygen therapy for critically ill patients: a meta-analysis of randomized controlled trials
Source: J Intensive Care. 2021 Jul 22;9:47. doi: 10.1186/s40560-021-00563-7 (PMC8295978; doi:10.1186/s40560-021-00563-7)
Supplement: Supplementary file 3 — Additional file 3. Table: Studies needed for full-reviewed but not included in the current meta-analysis. [file 40560_2021_563_MOESM3_ESM.docx]

**Additional file 3**

**Table S1:** **Studies needed for full-reviewed but not included in the current meta-analysis (n=6 trials)**

| No | Study | Reason of exclusion |
| --- | --- | --- |
| 1 | Sur MD, Namm JP, Hemmerich JA, et al. Radiographic sarcopenia and self-reported exhaustion independently predict NSIQR serious complications after pancreaticoduodenectomy in older adults. Ann Surg Oncol. 2015 Nov;22(12):3897-904. | Only 23% ICU admission |
| 2 | Vianna JR, Pires Di Lorenzo VA, Simões MM, Jamami M. Comparing the Effects of Two Different Levels of Hyperoxygenation on Gas Exchange During Open Endotracheal Suctioning: A Randomized Crossover Study. Respir Care. 2017 Jan;62(1):92-101. | Focused on effect of different oxygen strategies during open endotracheal suctioning |
| 3 | L Barrot, R Panwar, M Hardie, R Bellomo, G Eastwood, P Young, et al: Conservative versus liberal oxygenation targets for mechanically ventilated patients: pilot multicentre randomised trial. Intensive Care Medicine Experimental 2015, 3(Suppl 1):A423 | Only in abstract form as meeting report |
| 5 | Lång M, Skrifvars MB, Siironen J, Tanskanen P, Ala-Peijari M, Koivisto T, Djafarzadeh S, Bendel S. A pilot study of hyperoxemia on neurological injury, inflammation and oxidative stress. Acta Anaesthesiol Scand. 2018 Jul;62(6):801-810. | Focused on patients with traumatic brain injury |
| 6 | Zughaft D, Bhiladvala P, Van Dijkman A, et al. The analgesic effect of oxygen during  percutaneous coronary intervention (the OXYPAIN Trial). Acute Cardiac Care. 2013;15(3):63-68. | The aim was to investigate the analgesic effect of oxygen during percutaneous coronary intervention and to evaluate cardiac injury. |
