# Supplementary material for: Conservative oxygen therapy for critically ill patients: a meta-analysis of randomized controlled trials
Source: J Intensive Care. 2021 Jul 22;9:47. doi: 10.1186/s40560-021-00563-7 (PMC8295978; doi:10.1186/s40560-021-00563-7)
Supplement: Supplementary file 4 — Additional file 4. Fig: Definition of inclusion and exclusion criteria for patient population and regimens of conservative and conventional oxygen. [file 40560_2021_563_MOESM4_ESM.docx]

**Additional file 4**

**Table：Definition of inclusion and exclusion criteria for patient population and regimens of conservative and conventional oxygen**

| Study / Year | Inclusion criteria | Exclusion criteria | Oxygen regimen |
| --- | --- | --- | --- |
| Barrot et al^11^, 2020 | Patients were eligible for enrollment if they had undergone intubation and had been receiving mechanical ventilation for less than 12 hours for ARDS (defined according to the Berlin definition). | The main exclusion criteria were the use of long term oxygen therapy or noninvasive ventilation at home and cardiac arrest, traumatic brain injury, or cranial hypertension as the reason for hospitalization in the ICU. | Patients were assigned to either the liberal oxygen group (PaO_2_ target between 90 and 105 mmHg) or the conservative-oxygen group (PaO_2_ target between 55 and 70 mmHg) over the first 7 days of invasive mechanical ventilation or until extubation if the latter was performed earlier. |
| Yang et al^13^, 2018 | All patients aged 18 years or older and admitted to the ICU with an expected length of stay of 72 hours or longer were screened. | The exclusion criteria included the following: ICU readmission, patients with acute exacerbation of chronic obstruction disease, patients with severe acute respiratory distress syndrome [defined as PaO_2_/FiO_2_ <100 mmHg], inclusion in another interventional trial, a decision to withhold life-sustaining treatment, pregnancy, paraquat poisoning and not having been screened within 12 hours after admission. | In the low SpO_2_ group, the SpO_2_ target was 90–95%, with the FiO_2_ as low as possible. In the high SpO_2_ group, the SpO_2_ target was 96–100%, with FiO_2_ no lower than 30%. |
| Mackle et al^12^, 2019 | All adults (≥18 years of age) who were expected to receive mechanical ventilation in the ICU beyond the day after recruitment were eligible for inclusion in the trial. Enrollment was restricted to patients who had received less than 2 hours of invasive mechanical ventilation or noninvasive ventilation in the ICU. | Eligible patients who were not enrolled within the 2-hour time window were categorized as missed, rather than excluded, for the purposes of describing the enrollment of patients. | In the two groups, the default lower limit for oxygen  saturation as measured by pulse oximetry (SpO_2_) was 90%. In the conservative oxygen group, the upper limit of the SpO_2_ alarm was set to sound when the level reached 97%, and the FiO_2_ was decreased to 0.21 if the SpO_2_ was above the acceptable lower limit. In the usual-oxygen group, there were no specific measures limiting the FiO_2_ or the SpO_2_. |
| Asfar et al^2^, 2017 | Patients aged 18 years and older with septic shock who were on mechanical ventilation. | Exclusion criteria were severe hypoxaemia defined as PaO_2_:FiO_2_ < 100 mm Hg for a minimum PEEP of 5 cm H_2_O, plasma sodium concentration of less than 130 mmol/L or more than 145 mmol/L, intracranial hypertension, patient admitted for cardiac arrest, overt cardiac failure, under legal guardianship, no affiliation with the French health­care system, pregnancy, recent participation in another biomedical study or another interventional study with mortality as the primary endpoint, or an investigator’s decision not to resuscitate. | Patients were randomly assigned to the normoxia group (mechanical ventilation with FiO_2_ set to achieve an arterial haemoglobin oxygen saturation between 88% and 95%) or the hyperoxia group (mechanical ventilation with FiO_2_ of 1·0 for 24 h after inclusion; thereafter FiO_2_ as in the normoxia group. |
| Girardis et al^8^,2012 | All patients aged 18 years or older and admitted to the ICU with an expected length of stay of 72 hours or longer were considered for inclusion. | Exclusion criteria included age younger than 18 years, pregnancy, ICU readmission, a decision to withhold life-sustaining treatment, immunosuppression or neutropenia, and enrollment in another study. Because of a different protocol for oxygen supplementation, patients with acute de-compensation of chronic obstructive pulmonary disease and acute respiratory distress syndrome with a PaO_2_:FiO_2_ < 150 were also excluded. | Patients were randomly assigned to receive oxygen therapy to maintain PaO_2_ between 70 and 100mmHg or arterial oxyhemoglobin saturation (SpO_2_) between 94% and 98%(conservative group) or, according to standard ICU practice, to allow PaO_2_ values up to 150mmHg or SpO_2_ values between 97%and 100% (conventional control group). |
| Panwar et al^18^,2012 | ICU patients, aged ≥18 years, were eligible if they had been receiving invasive MV for <24 hours and their treating clinician expected MV to continue for at least next 24 hours. | Exclusion criteria included known pregnancy, imminent risk of death, or if the treating clinician lacked equipoise for the patient to be enrolled in this trial. | Following treatment allocation, the bedside nurse titrated the FiO_2_ within a range of 0.21 to 0.80 to achieve the assigned targets of 88-92% SpO_2_ for the conservative  oxygenation group or ≥96% SpO_2_ for the liberal oxygenation group. |
| Schjørring， 2021^19^ | Adult patients (≥18 years of age) who were admitted to the ICU with hypoxemic respiratory failure and who were receiving at least 10 liters of oxygen per minute in an open system or who had an FiO2 of at least 0.50 in a closed system; all the patients had placement of an arterial line and were expected to receive supplementary oxygen therapy for at least 24 hours in the ICU. | Cannot be randomized within 12 hours of icu admission; receives chronic mechanical ventilation; use of supplementary oxygen at home; previous treatment with bleomycin; organ transplant; withdrawal from active therapy or brain death deemed imminent; pregnancy confirmed by a positive urine or plasma human chorionic gonadotropin; carbon monoxide poisoning; cyanide poisoning; paraquat poisoning; methemoglobinemia; sickle cell disease; any condition expected to involve the use of hyperbaric oxygen treatment; consent not obtainable according to national regulations. | Patients received oxygen therapy targeting a Pao2 of either 60 mm Hg (lower-oxygenation group) or 90 mmHg (higheroxygenation group) for a maximum of 90 days. |
