# Supplementary material for: Conservative oxygen therapy for critically ill patients: a meta-analysis of randomized controlled trials
Source: J Intensive Care. 2021 Jul 22;9:47. doi: 10.1186/s40560-021-00563-7 (PMC8295978; doi:10.1186/s40560-021-00563-7)

**Additional file 5**

**Cochrane risk of bias**

**Risk of bias graph: review authors' judgements about each risk of bias item presented as percentages across all included studies.**


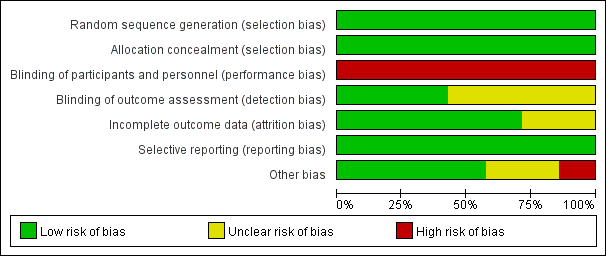


**Risk of bias summary: review authors' judgements about each risk of bias item for each included study.**


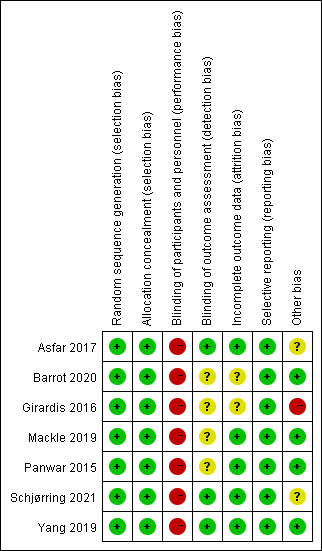

Supplement: Supplementary file 5 — Additional file 5. Fig: Cochrane risk of bias. [file 40560_2021_563_MOESM5_ESM.docx]
