# Supplementary material for: Conservative oxygen therapy for critically ill patients: a meta-analysis of randomized controlled trials
Source: J Intensive Care. 2021 Jul 22;9:47. doi: 10.1186/s40560-021-00563-7 (PMC8295978; doi:10.1186/s40560-021-00563-7)
Supplement: Supplementary file 6 — Additional file 6. Table: Adverse events. [file 40560_2021_563_MOESM6_ESM.docx]

**Additional file 6**

**Adverse events**

| Advise events | Barrot, 2020 | | Mackle, 2019 | | Asfar, 2017 | | Girardis, 2016 | | Panwar, 2015 | | Schjørring, 2021 | |
| --- | --- | --- | --- | --- | --- | --- | --- | --- | --- | --- | --- | --- |
|  | COS | COV | COS | COV | COS | COV | COS | COV | COS | COV | COS | COV |
| VAP | 17/99 | 22/102 |  |  |  |  |  |  |  |  |  |  |
| Arrhythmia | 23/99 | 16/102 |  |  |  |  |  |  |  |  |  |  |
| Septicemia | 11/99 | 19/102 |  |  |  |  | 11/216 | 22/218 |  |  |  |  |
| Seizure | 2/99 | 0/102 |  |  |  |  |  |  |  |  |  |  |
| Stroke | 4/99 | 1/102 | 0/484 | 1/481 |  |  |  |  |  |  | 19/1453 | 23/1457 |
| Delirium | 11/99 | 11/102 |  |  |  |  |  |  |  |  |  |  |
| Mesenteric ischemic | 5/99 | 0/102 |  |  | 4/217 | 7/217 |  |  |  |  | 32/1453 | 29/1457 |
| Pneumothorax | 6/99 | 10/102 |  |  | 5/217 | 5/217 |  |  |  |  |  |  |
| Hemoptysis | 6/99 | 1/102 |  |  |  |  |  |  |  |  |  |  |
| RRT | 10/99 | 10/102 | 94/484 | 108/481 |  |  |  |  |  |  |  |  |
| Digestive hemorrhage | 4/99 | 4/102 |  |  |  |  |  |  |  |  |  |  |
| Shock |  |  |  |  |  |  |  |  |  |  | 492/1453 | 521/1457 |
| Myocardial ischemia |  |  |  |  |  |  |  |  |  |  | 14/1453 | 8/1457 |
| Digestive ischemic |  |  |  |  | 9/217 | 5/217 |  |  |  |  |  |  |
| Hypoxemia |  |  | 2/484 | 0/481 |  |  |  |  |  |  |  |  |
| Atelectasis |  |  |  |  | 13/217 | 26/217 |  |  |  |  |  |  |
| Ventricular arrhythmias |  |  |  |  | 1/217 | 0/217 |  |  |  |  |  |  |
| ICU-acquired weakness |  |  |  |  | 13/217 | 24/217 |  |  |  |  |  |  |
| Nosocomial pneumonia | 17/99 | 22/102 |  |  | 32/217 | 30/217 | 30/216 | 37/218 |  |  |  |  |
| Nosocomial infection |  |  |  |  | 45/217 | 45/217 | 21/216 | 34/218 |  |  |  |  |
| New organ failure |  |  |  |  |  |  | 41/216 | 56/218 |  |  |  |  |
| Hemodynamic instability |  |  |  |  |  |  |  |  | 9/52 | 12/51 |  |  |
| New-onset ARDS |  |  |  |  |  |  |  |  | 11/52 | 11/51 |  |  |

ARDS=acute respiratory distress system; COS=conservative; COV=conventional; VAP=ventilator-associated pneumonia; ICU=intensive care unit; RRT=renal replacement therapy.
